# Portraying the developing PCK of Dutch pre-service geography teachers

**DOI:** 10.1080/10382046.2023.2281652

**Published:** 2023-11-24

**Authors:** Eefje Smit, Hanneke Tuithof, Tine Béneker

**Affiliations:** a Faculty of Geosciences, Utrecht University, Utrecht, The Netherlands; b Research Centre for Learning and Innovation, HU University of Applied Sciences Utrecht, Utrecht, The Netherlands; c Faculty of Humanities, Utrecht University, Utrecht, The Netherlands

**Keywords:** Geography education, PCK-development, PCK-portraits, PCK-survey, pre-service teachers

## Abstract

Pedagogical Content Knowledge (PCK) is used to describe the knowledge teachers use to teach a specific subject to a specific audience. Although PCK is linked to student success and motivation, relatively little is known about the PCK of geography teachers. Through a mixed methods approach, we surveyed a group of 73 Dutch pre-service teachers in their final year of geography teacher education. We used the PCK-consensus model to address both PCK-on action (teacher knowledge) and PCK-in action (teacher practice). We investigated the former through a CoRe-assignment and the latter through a quantitative survey. Teacher’s PCK-in action focussed on teacher-centred lessons with ample attention for visualisations, current events, and efforts to engage students. The results for PCK-on action confirmed the content dependency of PCK. Pre-service teachers chose different geographical topics and used different goals and strategies when teaching these topics. In this context, we also found that they experienced difficulties when teaching controversial issues. In a final step, we combined the results of both methods for 9 teachers in individual PCK portraits. These portraits show that coherence between PCK-elements and, therefore, PCK-quality is still weak for most pre-service teachers. Consequently, their fragile subject matter knowledge seems to influence their developing PCK.

## Introduction

In recent contributions to this journal, David Mitchell (2022) and Margaret Roberts (2023) argue for a more central role for values and powerful pedagogic practices in geography education. This raises the question to what extent these practices already take place in the geography classroom and whether they are also addressed in initial teacher education. In a previous study (Smit et al., 2023), we attempted to unravel geography teachers’ knowledge and practice by systematically reviewing empirical studies on Pedagogical Content Knowledge (PCK). PCK is the knowledge teachers use to teach a specific subject to a specific audience (Shulman, 1986). We chose PCK as a framework, because it is said to be predictive of teacher quality and, therefore, of student success (Coe et al., 2014) and motivation (Kunter et al., 2013). However, our review revealed that there is only a limited body of PCK-research on geography teachers at present. These studies were often self-reported and based on small target groups. Moreover, research on physical geography topics was overrepresented. Our review did give insight into common geography teaching practices, but only five studies gave detailed PCK-descriptions of geography teachers. Given the content and context dependency of PCK (van Driel & Berry, 2010), there is a need for more detailed insight into the knowledge and practice of geography teachers and the ways in which pre-service teachers develop this knowledge. This study is part of larger PhD research into the PCK-development of Dutch pre-service geography teachers. As a first step, we investigated the PCK of pre-service teachers. We ask with what PCK they start their teaching careers, using a mixed method research design to survey all Dutch pre-service geography teachers in the final year of their Bachelor of Education (BEd.) programme. This paper reports about the construction of this survey, its results, and its implications.

### PCK and PCK-development

The concept of PCK has known different conceptualizations over time. One of the most widely used is the conceptualization of Magnusson et al. (1999). They discern five PCK-elements: 1) teaching orientations (TO); 2) curriculum knowledge (CU); 3) knowledge of students’ understanding (SU); 4) knowledge of assessment (AS), and 5) knowledge of instructional strategies (IS). These elements underpin the more recent PCK-consensus model (Gess-Newsome, 2015), which regards PCK from a dynamic perspective in which there is an interplay between teachers’ PCK-on action (teacher knowledge) and PCK-in action (teachers’ classroom practice).

Several scholars acknowledge the influence of a teacher’s goals and beliefs on their teaching practice. According to the PCK-consensus model, these function as an amplifier or filter through which teachers mediate teacher actions. History teacher’s orientations have an even greater steering role in teaching practice as Tuithof (2017) found. An interesting example of this role in geography education is provided by Campbell et al. (2017) who portrayed Earth Science teacher Max as having different goals and using different teaching approaches for different geographical topics. Seow (2016) found that power relationships in the teaching contexts of pre-service teachers were even more influential. She stresses the importance of addressing professional identities in teacher education, because pre-service teachers with well-developed teacher identities were better able to pursue teaching practices that were meaningful to themselves.

For all five PCK-elements, our review study (Smit et al., 2023) presents descriptions of geography teacher’s PCK (a summary can be found in Appendix A). Apart from that, we also found descriptions of constraints and challenges geography teachers experience, among others a lack of PCK and of Subject Matter Knowledge (SMK).

Although the separate PCK-elements give insight into the knowledge and practice of geography teachers, they do not elicit their PCK-quality. The coherence between PCK-elements is probably an indicator of this (for example Barendsen & Henze, 2019; Magnusson et al., 1999). Experienced teachers with high PCK-quality are likely to have strong coherence between PCK-elements. Their teaching practice is aligned with all PCK-elements. PCK-development is, therefore, seen as a transition from fragmented to more integrated PCK (Aydin & Boz, 2013). SMK and teaching experience are considered important prerequisites for the development of PCK (van Driel & Berry, 2010). And pre-service teachers need to develop knowledge in all PCK-elements with respect to all topics they teach (Magnusson et al., 1999).

From our prior review study (Smit et al., 2023), we learned that coherence between PCK-elements is best visible in elaborate descriptions of individual teacher’s PCK (in this study referred to as PCK-portraits). In these portraits, we found examples of coherence between the elements 1) teaching orientations; 5) instructional strategies, and 3) knowledge of students’ understanding. Clausen (2018), for example, reports about four Danish teachers whose enacted PCK seemed to be aligned with their diverse teaching orientations and beliefs. Moreover, Australian pre-service teachers in Reitano and Harte (2016) demonstrated that they were aware of their students’ understanding in their lesson planning. There were, however, considerable variations between these teachers. Our review also found PCK-portraits which lacked coherence between the same PCK-elements (Lane, 2009; Mphathiwa, 2015). These portraits were from teachers with either little teaching experience or with a lack of SMK.

### PCK research

Investigating PCK-on action requires a different approach than investigating PCK-in action. PCK-on action is often examined through (focus group) interviews (for example Clausen, 2018; Reitano & Harte, 2016). Loughran et al. (2004) developed Content Representations (CoRe) for research into PCK-on action. A CoRe is a standardized framework with questions comprising all Magnusson’s PCK-elements which can be used as guidance for an interview or an open-ended assignment. Research which examines PCK-on action through quantitative methods such as surveys or PCK-tests has increased over recent years. For example, Rosenkränzer et al. (2017) used a PCK-test to assess pre-service geography teachers’ PCK-development, particular for systems thinking. Similar domain-specific knowledge tests on PCK and SMK were developed by Schilcher et al. (2021).

When investigating PCK-in action, researchers mostly rely on lesson observations or the use of (video) stimulated recall. A specific method for eliciting PCK-in action are Personal and Pedagogical-experience Repertoires (PaP-eR) as proposed by Loughran et al. (2004). The purpose of a PaP-eR is to let teachers reflect on their PCK-in action. PaP-eRs do not have a fixed format, but can, for example, consist of a log book, an analysis of student materials, or annotated videos. Finally, Barendsen and Henze (2019) developed a quantitative lesson observation scheme that makes it possible to tally teacher actions every minute.

In summary, PCK-on action and PCK-in action have both been investigated through quantitative and qualitative means. Qualitative methods can result in more in-depth and personal PCK descriptions, but they are time consuming and are therefore often carried out on small target groups. The qualitative studies in our review, for example, had between 1 and 20 participants. Nevertheless, Brooks (2006) and Puttick (2016) argue for extended interviews with open-ended questioning when researching teacher knowledge and practice, since “teachers express what they do and their knowledge through narratives” (Brooks, 2006. pp. 356). Knecht et al. (2020), on the other hand, make a stand for the use of quantitative methods on larger target groups, since “disunity of qualitative inquiry and small sample sizes compromise the statistical power of research findings and hamper generalization and development of robust theories of geography education” (pp. 470). This is in line with Bednarz and Heffron (2014) who argue that larger-scale and more rigorous research is required that focusses on teaching geography as well as to foster the development of high-quality geography teachers. Considering this debate, Evens et al. (2015) recommend the use of a mixed method research design to investigate PCK.

### Characteristics of Dutch geography teachers

Since PCK is known to be context dependent, we will give a short impression of current research on Dutch geography teachers. In this body of research, a number of themes emerge. First, geography text books are seen as an important resource for lesson planning (Krause et al., 2017, 2021; Pauw, 2021). Secondly, there seems to be a focus on lower order thinking skills in Dutch geography lessons. This is possibly due to the lack of assignments on higher order thinking skills in text books (Krause et al., 2022) and in national exams (Bijsterbosch et al., 2016). This focus on lower order thinking skills was also reported by Hooghuis et al. (2014), who found that Dutch geography teachers (*n* = 307) most frequently used strategies that focus on basic geographical knowledge. That is, they used geographical concepts and sources with geographical information such as maps, graphs, and photographs. There was little use of more complex and integrative assignments such as opinion forming or analysis of regions or spatial problems. According to Pauw (2021), Dutch geography teachers experience difficulties when addressing futures thinking. The open kind of teaching and learning required for envisioning the future appeared to be difficult for both students and teachers. Recent research by Duindam et al. (submitted) addresses the challenges geography teachers are dealing with when teaching controversial issues. Although many teachers hope that their teaching will lead to sustainable transformation, they hesitate to pose such goals directly to their students. Finally, (Krause et al., 2021) found that pre-service teachers experience difficulties related to time management, the heterogeneity of students, and again higher order thinking.

Taking into account what we know about Dutch geography teachers, PCK (-development) and PCK research, we defined the following research questions aimed at better understanding the developing PCK of Dutch pre-service geography teachers.

- What characterises Dutch pre-service geography teachers’ (*n* = 73) PCK-in action?
- How is content-dependency visible in their PCK-on action on different geographical topics?
- To what extent is coherence between elements present in the PCK of individual pre-service geography teachers?

## Methods and materials

**Figure 1.**
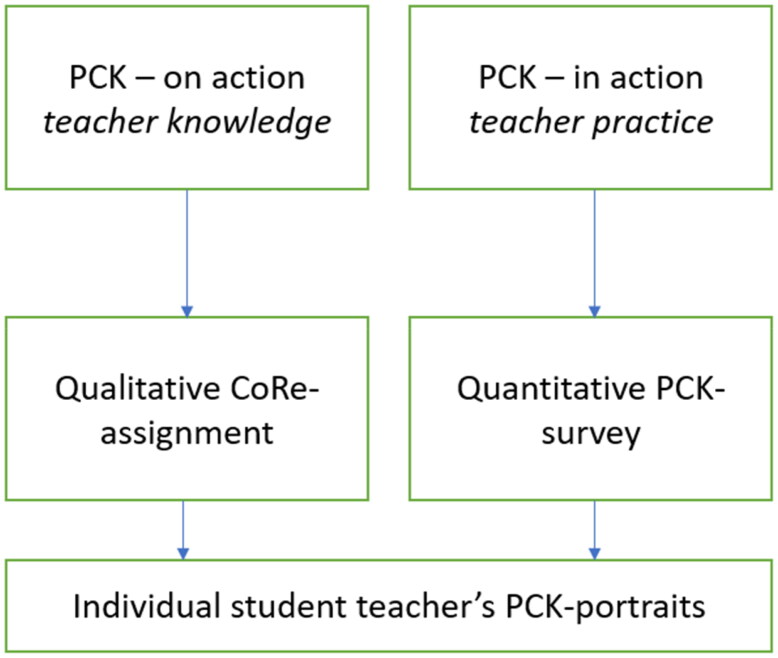
Research design.

Considering the aforementioned debate on quantitative and qualitative methods, we followed Evens et al. (2015) recommendation and chose a mixed method research design for our study (Figure 1). We favoured this approach for it enabled us to survey a larger target group, without abandoning the opportunity of respondents elaborating on their answers.

### Investigating PCK-in action

We developed a quantitative survey to measure PCK-in action (available on request). This survey uses Magnusson’s PCK-elements as a framework, to which we added a category of challenges and constraints experienced by teachers. Each category was subdivided according to the outcomes of our PCK-review (Smit et al., 2023). For example, category 4) knowledge of assessment was subdivided in: written test; non-written test; formative evaluation by teacher; formative evaluation by peer; formative evaluation by self or other. Pre-service teachers were asked whether they had applied any of these activities in their classroom practice in the past 6 months. Frequencies were measured on a five-point scale (never - in one lesson - in some lessons - in almost every lesson - in every lesson), adapted from (Hovey et al. (2019). Some categories in our review were not suitable for use in a survey. For example, the category of 1) teaching orientations was very broad. We decided to survey this category by asking teachers about their teacher identity and their geography perspectives. These teacher identities were derived from Seow (2016), that is, geography teacher, teacher who happens to teach geography (in this study referred to as pedagogue), and geographer. And since we were interested in school geography goals, we also asked teachers to rank their Top 3 geography perspectives for which we used a set of ten geography perspectives (Table 1) as proposed by Knecht et al. (2020), who adapted these from Catling (2004).

**Table 1.** Geography perspectives (Knecht et al., 2020).

Perspective	Purpose of geography/geography teaching
Globalist	To orient oneself in the world. To get to know the world. To develop general knowledge of the world.
Earthist	To understand the phenomena and processes that occur on Earth; how things work; how the Earth works.
Interactionist	To reveal relationships and interdependence between humans and nature/the environment
Placeist	To get to know different places and activities of the people who live there
Environmentalist	To warn about environmental crises. To pursue sustainability. To seek solutions for environmental crises.
Localist	To orient oneself in one’s immediate surroundings
Locationalist	To determine the location. To know where the place is located and what is found in the particular location/region.
Map-lover	To analyse, interpret, and create maps
Synthesizer	To interconnect subject matter and perspectives on different disciplines within geography and beyond
Facilitator	To develop practical or generic knowledge and abilities through geography.

Furthermore, we subdivided the subcategory “strategies to facilitate student learning,” because of our interest in the specific strategies (e.g. visualisation, differentiating etc.) these pre-service teachers applied. For this subdivision, we again used the data underpinning our review study.

Constructing this PCK-survey happened in various rounds. Pre-service teachers outside the target group gave feedback on a first draft in which they tested the frequency scales. A geography education expert and an expert in assessment both provided feedback in a next round. This helped us to clarify the answering categories and to avoid misinterpretation. A final draft was tested by six pre-service teachers outside the target group. They tested the online survey tool and pointed out spelling errors and “vague” descriptions.

Data was collected through an online survey tool in which data can be stored anonymously (the process of data collection is further described below). After collection, we analysed the data from this quantitative PCK-survey by addressing weightings to the frequency scales (1 = never to 5 = in every lesson). By doing so, we were able to calculate averages and standard deviations for every category and subcategory.

### Investigating PCK-on action

To investigate pre-service teacher’s PCK-on action, we used an assignment based on the CoRe-framework (available on request). We used the version which Tuithof (2017) adapted from Loughran et al. (2004). Pre-service teachers were asked to complete the assignment for a topic they preferred. In our experience, most teachers have a preference for either physical or human geography. With our choice of migration and plate tectonics, we tried to comprise human geography and physical geography respectively. With our choice of natural hazards, we aimed to foster teachers preferring the human-environment relationship.

Data was collected through the online survey tool mentioned earlier. We analysed this data by means of descriptive coding (Saldaña, 2021), for which the categories and subcategories in the quantitative survey functioned as a framework. A code book is available on request. For each topic, one CoRe-assignment was also coded by another researcher to check reliability. The main discussion focused on the subcategory “strategies to facilitate student understanding.” We decided to categorize these under 5) instructional strategies.

### Investigating coherence between PCK-elements

In a final step we combined, for 9 individual students, data of the quantitative PCK-survey with the results of the qualitative CoRe-assignment into individual PCK-portraits. For each topic, we chose the three most elaborate CoRe-descriptions (e.g. largest number of codes). We performed a within-case analysis on these nine portraits to reveal coherence or a lack of coherence between PCK-elements. When, for example, a teacher with an Earthist perspective on geography education used experiments and drawings to clarify processes, we classified this as coherence between PCK-element 1) teaching orientations and 5) instructional strategies. Lack of coherence was classified when a teacher, for example, was not able to transfer his/her geography perspective into related teaching strategies. The analysis of PCK-portraits was done by the first author and then discussed with the second author until the analyses reflected the portraits comprehensively.

### Data collection & target group

The pre-service teachers in our sample all follow a Bachelor of Education (BEd.)-programme at a Dutch University of Applied Science. This qualifies them to teach geography or humanics in the lower levels of secondary education (12–15 years). In order to teach in the higher levels, they have to obtain an additional Master’s degree. There are eight institutions who offer BEd.-programmes for geography in the Netherlands. All institutes have to work towards the same national standards (Dutch: bekwaamheidseisen) (Rijksoverheid, 2017). The BEd.-curriculum comprises 4 years of which approximately 50% is dedicated to SMK and PCK, 25% to school-based internships, and 25% to professional and personal development (Van Kempen et al., 2016). This curriculum is underpinned by a subject matter and subject pedagogy knowledge base^1^(Vereniging Hogescholen, 2017). Considerable consultation takes place between the institutes, for example, through an annual peer review in which institutions audit each other’s curriculum. Students in the BEd.-programme often start immediately after their own secondary education, which means that pre-service teachers are typically between 17 and 19 years old when they start their teacher training. Considering that teaching experience and SMK are important prerequisites for PCK-development (van Driel & Berry, 2010), note that these pre-service teachers start their teacher education without any teaching experience and that their SMK is limited to what they learned in secondary education.

The first author collected data in the spring of 2022. She visited five education institutions in person and two online; students of one institution were approached by e-mail. Most pre-service teachers completed the online survey in the presence of the first author. Our survey had 73 respondents out of a total of 117 pre-service geography teachers in their final year (62%). Respondents were asked for their informed consent prior to participating in this study.

## Results

This section describes successively the results of the PCK-survey (PCK-in action), the CoRe-assignment (PCK-on action), and individual teachers’ PCK-portraits (coherence between PCK-elements).

### PCK – in action

Our survey asked pre-service teachers about their PCK-in action in the preceding 6 months. A summary of the quantitative results are available as online supplement. The results presented here give insight into pre-service teachers classroom behaviour for school geography in general.

**Figure 2.**
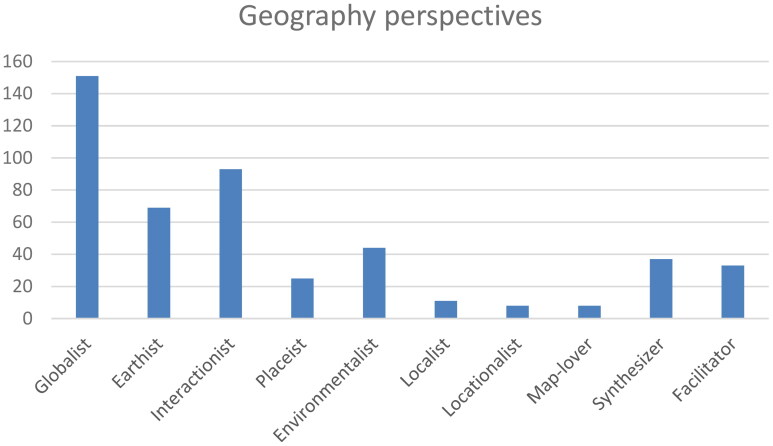
Teacher orientations expressed as geography perspectives. The scores represent the average values of the ranking assignment (score 3 for Rank 1, 2 for Rank 2, and 1 for Rank 3).

Next to classic geography perspectives such as Globalist, Earthist, and Interactionist perspectives, the 73 pre-service teachers ranked perspectives related to interdisciplinarity, sustainability, and the facilitation of practical knowledge relatively highly (Figure 2).

PCK-in action consisted mainly of teacher-led presentations and general class discussions, which resembles the results in our review study (Smit et al., 2023). Pre-service teachers often used movies, pictures, and stories in their lessons. Nevertheless, student activities remained at a lower cognitive order: teachers focused on describing concepts and using concepts in context. Textbooks were a main source for lesson planning, although there is substantial variation between teachers. Both findings are consistent with what we know from earlier research on Dutch geography teachers. The pre-service teachers in our study appeared to be committed to their students. They reported that they facilitate learning through visualization and connect geographical topics to current events and their students’ personal lives. They also present learning goals and use everyday language. Formative evaluations by teacher also appeared to be common practice. Test items in written tests tended to focus on facts and concepts. Finally, the pre-service teachers in our sample reported that they occasionally lack SMK, PCK, and resources.

### PCK - on action

The 73 pre-service teachers in our sample completed a CoRe-assignment on a geographical topic they preferred. Migrations as a topic was mainly chosen by teachers with a pedagogue identity (12 of *n* = 26), and plate tectonics by teachers who identified with geographers (9 of *n* = 19). Natural hazards was favoured by both geography teachers and pedagogues (both 9 of *n* = 25). Most pre-service teachers gave short answers without much detail. It was not always clear what they meant (an example of this is “let students think about the topic” (Migration, Case 22)). These quotes were then coded as “other.” The results of these CoRe-assignments (summarized in Table 2) show interesting differences between their geography perspectives and teaching practice for each of the topics. Nevertheless, there were no notable differences within PCK-element 4) knowledge of assessment. We have therefore chosen to omit this PCK-element in this section.

**Table 2.** Summary of results regarding PCK-on action.

	Natural hazards (*n* = 25)	Plate tectonics (*n* = 19)	Migration (*n* = 26)
Teaching Orientations	Interactionist Globalist Environmentalist	Earthist Globalist	Facilitator Globalist
Curriculum Knowledge	Knowledge goals	Knowledge goals	Knowledge goals Skill goals Attitude goals
Student Understanding	Prior knowledge and skills Engaging students	Prior knowledge and skills Engaging students	Student diversity Student attitudes
Instructional Strategies	Lecture Use of visualisations Inquiry Based-Learning Current Events	Lecture Use of visualisations Drawings Simulations	General Class Discussion Use of visualisations

Pre-service teachers who chose migration set goals relating to the facilitator perspective in which children learn generic and practical knowledge through geography. Apart from knowledge, they set skills and attitude goals for their lessons. Skill goals related mainly to opinion forming or reasoning. The attitude goals referred to diversity and to the goal “have (maybe) a better understanding of the situation of others” (Migration, Case 3). These teachers primarily used general class discussions and incorporated movies and pictures into their classes. Pre-service teachers took into account the various backgrounds of their students and occasionally of themselves, which is typical of the topic of migration: “I often use my student’s knowledge on the topic, for example a student with a migration background who wants to share his story. His fellow students will learn a lot from that.” (Migration, Case 22). That the topic of migration is controversial and therefore a challenging teaching subject was mentioned by almost all of these pre-service teachers whether in cities with a large cultural diversity or in those rural areas that consider migration negatively. Some pre-service teachers even suggested “avoiding the topic” as an option, because “students can (…) experience negative feelings” (Migration, Case 2).

Pre-service teachers who chose plate tectonics handled this topic mainly from an Earthist and Globalist perspective, in which they focus on earth’s processes and on general knowledge of the world. They primarily set knowledge goals for their lessons. Skill goals were absent. Their teaching practice consisted for a large part of lectures, in which they often used movies and pictures: “For example, drawing, using lots of images or watching a video” (Plates, Case 15). Drawings were mentioned frequently, but it was not always clear who did the drawing - the teacher or the student - and what the purpose of this drawing was. These teachers refer to the topic as abstract and difficult to students, without further elaborating. They do take an effort in engaging their students; some say this is easy since “students are very curious about this topic” (Plates, Case 10). Others mention that it is difficult to engage students, because “students are not very interested. They think it is boring and not fascinating” (Plates, Case 3). They use many visuals, link the topic to current events, and use differentiation all of which to facilitate learning. “We do this by making drawings and simulations. In this way the invisible processes are easier to understand. Besides it helps to give examples (…) preferably things they recognize from their own surroundings or the news” (Plates, Case 12).

Finally, pre-service teachers who chose the topic of natural hazards did this mainly from an Interactionist, Globalist, or Environmentalist perspective. They aim to reveal relationships between human and nature and connect natural hazards to climate change. They set almost only knowledge goals for their lessons. By doing so, they referred to causes and effects of natural hazards, but (preventive) hazard management was almost absent: “how natural hazards can occur, what causes them, and what the effects for the local community are” (Hazards, Case 20). The teachers in our sample mainly treated this topic from a physical geography perspective. These teachers reported the use of lectures with movies and pictures, but also mentioned inquiry based learning. For instance, they asked their students to “investigate the effects of a natural disaster and make a poster out of it” (Hazards, Case 12). These teachers took into account their students’ prior knowledge as well and took an effort in engaging them. They did so by using visuals, linking the topic to current events, and through differentiating. A pre-service teacher remarked: “if a natural hazard has just happened, I pay extra attention to it” (Hazards, Case 17).

### Coherence between PCK-elements

Through our analysis of the PCK-portraits of 9 individual teachers, we were able to assess their PCK-quality. Within these 9 portraits we found pre-service teachers with more or less elaborate PCK. Table 3 shows portraits of a teacher with elaborate PCK (Plates 3) and of a teacher with developing PCK (Migration 3). The coherence between their PCK-elements is visualized in the final row. Plates 3 clearly shows more coherence between elements than Migration3 and is therefore assumed to have higher PCK-quality.

**Table 3.** PCK-portraits with descriptions of PCK-elements for two pre-service geography teachers. This portrait is based on both PCK- on action and PCK-in action.

	Teacher Plates 3	Teacher Migration 3
Teaching Orientations	Teacher identity: geography teacher Geography perspectives: Globalist Placeist Interactionist Geographical relevance: “they read on the news about earth quakes and volcanic eruptions, they see pictures of Hawaii and Iceland. All current events which are actually related to plate tectonics.”	Teacher identity: pedagogue Geography perspectives: Globalist Facilitator Earthist Geographical relevance: “they will be able to explain things they can see in their own surroundings.”
Curriculum Knowledge	Uses: text books, national curriculum, school curriculum. Geographical content: “that the earth crust is actually a jigsaw, with pieces moving around and what the effects and consequences of these movements are.”	Uses: national curriculum, sequence of topics, text books, and own interest. Geographical content: “why people migrate. What the consequences of migration for a country are.”
Knowledge of student’s under­-standing	Takes into account: prior knowledge, attitudes and student engagement. Through: presenting learning goals, connect to current events, use of visualisations, and everyday language. “There are a lot of misconceptions with students.” “Students first have to understand how earth is build up and how it works, and only then can you start with plates and plate movement. (…) For students it can sometimes be overwhelming.” “when I teach this topic to a lower grade, it will be totally different.”	Takes into account: student engagement and different backgrounds. Through: presenting learning goals, repeating lessons, everyday language, and active learning. “I often use the knowledge of students, for example a student who migrated and wants to share the story.” “in lower grades I use easier language.”
Knowledge of assessment	Assesses knowledge and skills in written tests. And formative evaluation occasionally: “asking a lot of questions during the lesson” and use of exit tickets with check questions.	Through written tests, mostly developed by experienced colleagues. And formative evaluation rarely: “by checking lesson goals at the end of the lesson. By asking questions about the topic.”
Knowledge of instructional strategies	Uses presentation, general class discussion. Appears to have a lot of variation in lessons. With the plate tectonics topic: “I always handle this topic extra slowly.” “I show lots of pictures and let students talk about them to foster their thinking. I use video’s with questions (…) then they have to focus well and think at the same time.” And use of experiments, in which students are actively engaged and can “see with their own eyes what the difference is between an oceanic and continental plate.”	Mainly use of presentation, next to other strategies. With the migration topic: presentation, “movies on migrants who came to the Netherlands,” using lots of pictures and “let students think about the topic”
Visualisation^2^	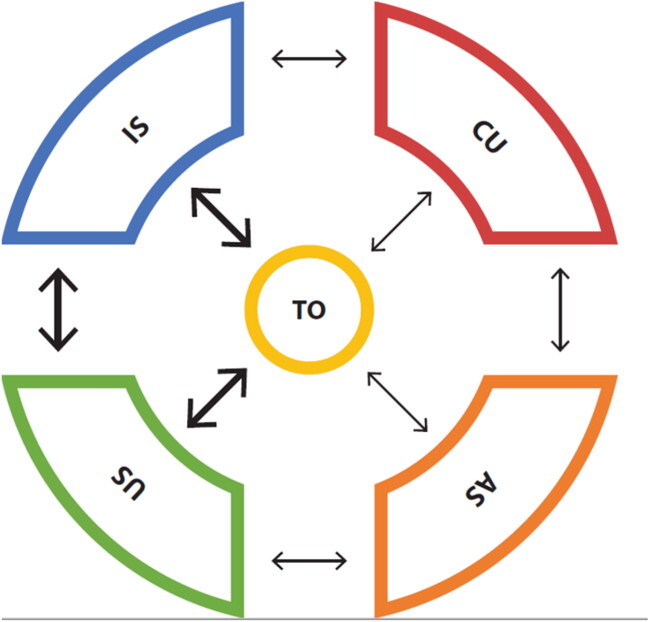	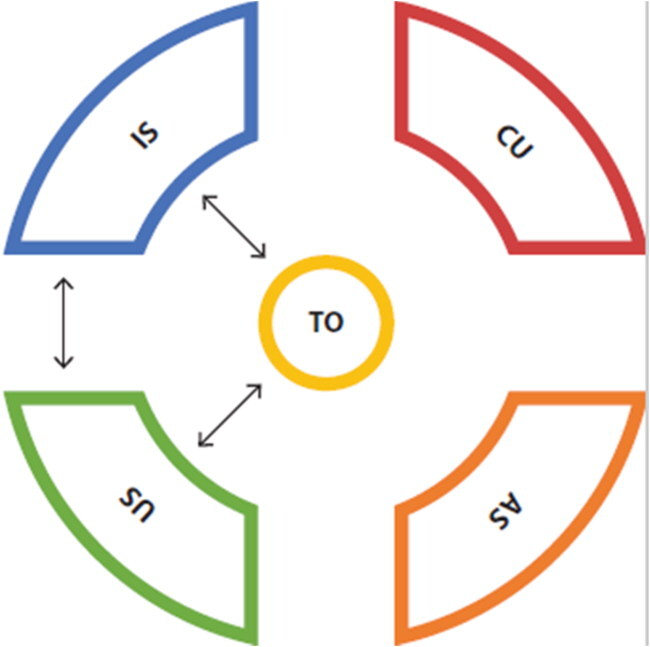

When examining the portrait of Plates 3, it stands out that this teacher takes into account knowledge of student’s understanding of the topic throughout the teaching practice. The teacher is aware of learning difficulties related to the topic and is able to provide students with a variety of appropriate teaching strategies. When teaching this topic to a lower grade, (s)he would use easier language and a different teaching approach. This teacher gains insight into possible misunderstandings by asking questions throughout the lesson. Finally, the identity of geography teacher is both reflected in the chosen geography perspectives as in the balance between teaching content and adapting to student’s needs.

The other portrait, teacher Migration 3, shows less coherence. This teacher’s identity of pedagogue is reflected in the geography perspective of facilitator and in the way in which this teacher wants to engage students. The teacher relates the topic of migration to students’ various backgrounds and adapts his/her language to the audience. However, this teacher appears not to be aware of learning difficulties the students might have and does not adapt teaching strategies to this topic specifically. The local surroundings, which are seen as an important learning goal, have no follow up in the teaching strategies. Assessment of knowledge seems to happen only at lesson ends or in written tests; therefore, it will not be possible to adapt teaching strategies during the lesson. When teaching this topic to a lower grade, the teacher suggests that “easier language” can be used.

Altogether, our results show considerable variation in PCK-quality between pre-service teachers. Apart from the differences in coherence, we also found a particular school context or fellow colleagues to be quite influential on their teaching practice. Migration 2 mentions “my teaching practice is momentarily influenced by my colleagues. I do mainly what they do, because they have done this for some time.” This influence leads to an internal conflict: “I personally (…) prefer a game or a cooperative assignment, but it didn’t happen.” Another teacher finds this less troubling, although the school context is recognizable throughout the PCK-portrait. Plates 2: “at the moment I teach at an inquiry-based/anthroposophical school. Here you have to make your students wonder in order to raise learning questions based on intrinsic motivation. I don’t give them the knowledge about plate tectonics, I give them resources to construct this knowledge themselves.”

## Discussion

Our study shows that pre-service teachers have developed knowledge in each PCK-element at the end of their teacher education training (Magnusson et al., 1999). It is remarkable that major variations were found in PCK-on action between different geographical topics. Teachers with different teacher identities chose different geographical topics. They set different geography goals, handled different teaching strategies, and experienced different challenges with each topic. This content-dependency was earlier addressed by Campbell et al. (2017) for the subject of earth sciences and corresponds with what we know about PCK in general (van Driel & Berry, 2010). Apparently, the broad knowledge base of geography requires broadly developed PCK. For geography teacher education, it can imply a need to focus on topic-specific PCK within the subject of geography.

Regarding PCK-quality, we found that coherence between PCK-elements is still weak for most pre-service teachers. Their goals with the school subject remain vague, and they are not always able to adapt teaching strategies to their students’ learning difficulties. In this light, it is remarkable that these pre-service teachers already pay so much attention to complex strategies such as formative assessment or differentiating. It appears that they perform these techniques almost instrumentally, without realising that the outcomes are possible resources for their teaching. Instead, special attention should be paid to the teaching of controversial topics such as migration, in teacher education. Pre-service teachers clearly faced difficulty teaching these issues and relating them to their own values. This corresponds with the findings of Biddulph et al. (2020), Pauw (2021), and Mitchell’s (2022) earlier argument. The fragile SMK of these pre-service teachers likely contributes to insecurities in teaching controversial issues.

### Limitations

Our research instruments (CoRe-assignment and quantitative survey) enabled us to acquire a general overview of pre-service teachers’ PCK for a larger target group. Our data made it possible to describe generic teaching practices, such as general class discussion or the use of visualisations. However, based on the same data, it was not possible to tell how pre-service teachers enacted these practices on a micro level. For example, how did a class discussion take place? What were the questions asked by the teacher? What part of the class was actively engaged in the discussion? And how did the teacher react to questions from students?

Although the CoRe-assignment did provide more in-depth insight into teacher knowledge, most pre-service teachers gave short and incomplete answers. The first author was present when they completed the assignment, but still most CoRe-assignments fell short of micro-level PCK-descriptions. This may have resulted in an underestimation of their actual PCK. A follow-up interview would probably have resulted in more elaborate and complete answers thus providing better insight into their PCK-on action.

## Further research

This study portrayed the developing PCK of Dutch pre-service geography teachers. It stresses once more the influence of contextual factors such as the school context, the text book used, or their school-based mentor (as earlier illustrated by Seow, 2016). In order to fully understand PCK-development, we need to know more about these factors and about the extent to which they influence the PCK of pre-service geography teachers. This PhD-research will continue to explore the PCK-development of BEd-students by following a group of pre-service teachers during their geography teacher education.

## Supplementary Material

Supplemental Material

## Notes

1 In the Netherlands, subject pedagogy is traditionally related to the German concept *Fachdidaktik*.

2 Bold arrows refer to strong coherence, thin arrows to weak coherence.
