# Supplementary material for: Portraying the developing PCK of Dutch pre-service geography teachers
Source: Int Res Geogr Environ Educ. 2023 Nov 24;33(3):177–92. doi: 10.1080/10382046.2023.2281652 (PMC11325438; doi:10.1080/10382046.2023.2281652)
Supplement: Supplemental Material [file RGEE_A_2281652_SM6712.zip › New folder/Appendix_A_Elements_of_geography_teacher_s_PCK.docx]

Appendix A Elements of geography teacher’s PCK

| PCK-element (Magnusson et al., 1999) | Examples of geography teachers’ PCK (Smit et al., 2023) |
| --- | --- |
| 1. Teaching orientations | - School geography goals - Approaches to learning - Beliefs about specific geography teaching strategies - Beliefs about the nature of geography |
| 1. Knowledge of curriculum | - Curriculum emphasis - Curriculum influences |
| 1. Knowledge of students’ understanding | - Students’ prior knowledge and skills - Students’ alternative conceptions - Students’ attitudes on geographical topics |
| 1. Knowledge of assessment | - Formative assessment - Graded tasks - Written tests - Specific geographical test items |
| 1. Instructional strategies | Teacher activities:   - General class discussion - Using resources - Fieldwork - Lecture - Inquiry-based learning - Strategies to facilitate student learning   Student activities:   - Describing/relating concepts - Analysing maps, graphs, data - Using concepts in context |
| Constraints and challenges experienced by teachers | - Lack of PCK - Lack of Subject Matter Knowledge (SMK) - Lack of time and resources |
