# Supplementary material for: Portraying the developing PCK of Dutch pre-service geography teachers
Source: Int Res Geogr Environ Educ. 2023 Nov 24;33(3):177–92. doi: 10.1080/10382046.2023.2281652 (PMC11325438; doi:10.1080/10382046.2023.2281652)
Supplement: Supplemental Material [file RGEE_A_2281652_SM6712.zip › New folder/Online supplement Summary PCK-in action.pdf]

Summary of results for PCK-in action.

Scores represent frequencies: 0 = never, 3 = in some lessons, 5 = in every lesson.

Online supplement to: Smit, E., Tuithof, H. & Béneker, T. (2023) Portraying the developing PCK of Dutch pre-service geography teachers

PCK-Element	Average	StDev
**Instructional Strategies: Teacher Activities**		
Lecture	3,91	0,79
General Class Discussion	3,4	0,8
Inquiry Based Learning	2,78	0,85
Fieldwork	1,46	0,72
Practical Work	1,94	0,85
Use of geographical resources	3,29	0,74
Other	2,19	1,26
**Instructional Strategies: Student Activities**		
Describing/Relating concepts	3,68	0,77
Use of Concepts in Context	3,65	0,78
Use of photo's, movies, stories	3,84	0,73
Use of maps and data	3,14	0,57
Analysis of Regions	2,79	0,9
Analysis of (spatial) problems	2,75	0,91
Problem Solving	2,85	0,88
Futures Thinking	2,94	0,75
Opinion Forming	2,48	0,88

Map Production	1,81	0,84
Other	1,7	1,1
**Instructional Strategies (strategies to facilitate student learning)**		
Use of everyday language	4,2	0,83
Use of visuals	4,1	0,75
Active Learning	3,84	0,78
Connecting to everyday life	3,84	0,76
Repeating Lessons	3,52	1,01
Present lesson goals	4,06	1
Differentiating	3,4	0,91
Relating to current events	3,75	0,77
Give students choice	3,28	0,85
Use scaffolding	2,66	1,21
Use of checklists	2,66	1,23
Use of personal stories	3,15	0,86
Experiential learning	2,03	1,01
Other	1,13	0,33
**Knowledge of Student Understanding**		
Prior knowledge and skills	4	0,69
Engaging Students	3,74	0,8
Student Attitudes	3,49	0,98
Student Diversity	3,38	1,07
Alternative Conceptions	3,44	0,91
Other	1,5	1,02

**Knowledge of Assessment**		
Formative by teacher	3,13	0,92
Written test	2,86	0,79
Non-written test	2,55	0,84
Formative by peer	3,07	0,92
Formative by self	1,88	1,06
Other	2,6	1,54
**Curriculum influences**		
Text Books	3,84	1,03
Teacher Interest	3,26	0,93
Student Interest	3,2	0,86
National Standards	3,32	1,26
Geography Department	2,96	1,33
School Curriculum	2,88	1,4
National Exams	2,49	1,39
Sequence of Topics	3,1	1,23
Mentor	1,7	1,11
Other	1,2	0,6
